# ﻿Citizen science reveals a shift in the commonness and rarity of Trichoptera in the Netherlands

**DOI:** 10.3897/zookeys.1263.147805

**Published:** 2025-12-10

**Authors:** David Tempelman, Wilco C. E. P. Verberk, Maria J. Sanabria

**Affiliations:** 1 Semblis Foundation, Bisschop Zwijsenplein 26, Vught, Netherlands Semblis Foundation Vught Netherlands; 2 Department of Ecology, Radboud Institute for Biological and Environmental Sciences, Radboud University Nijmegen, Nijmegen, Netherlands Radboud University Nijmegen Nijmegen Netherlands

**Keywords:** Aestivation, citizen science, climate warming, diapause, drought, Trichoptera

## Abstract

Understanding aquatic insect species responses to environmental change necessitates robust data on species occurrences. Using over 140,000 citizen-science records from the Netherlands, we analysed trends in adult caddisflies (Trichoptera) to explore temporal changes in 146 species with differing life-history traits, particularly summer diapause. Diapause, a state of suspended development, allows certain Trichoptera to withstand unfavorable environmental conditions. Our analyses indicate a disproportionate rise in observations of species with summer diapause, which is independent of ease of detection (i.e. a species’ body size and rarity). This is exemplified by the increased prevalence of the caddisfly, *Glyphotaelius pellucidus* (Retzius, 1783), a species with an adult summer diapause. We interpret these results from the perspective of climate change. Summer droughts and high water temperatures have become more prevalent in recent decades, representing harsh conditions for aquatic larvae. Having an adult summer diapause helps species to avoid these harsh conditions, which is consistent with the observed changes. Conversely, species lacking summer diapause, such as *Mystacides longicornis* (Linnaeus, 1758), may be more vulnerable to warming. Our results underscore the value of citizen-science data in elucidating shifts in insect populations and offer insight into the adaptive significance of life-history traits.

## ﻿Introduction

Climate warming affects ectothermic organisms, including insects, in a myriad of ways. Direct effects of warming include accelerated physiological processes such as growth and development, while indirect effects may occur via changes in precipitation, leading to droughts and fires (Harvey et al. 2023). Changes in the environment will lead to shifts in the commonness and rarity of species. Linking such shifts in species occurrences to climatic differences across space and time requires data to be collected with sufficient temporal and spatial resolution. In this paper, we use citizen-science data of Trichoptera to gain insight in potential changes their commonness and rarity in the Netherlands.

One way in which we can comprehend variation in commonness and rarity among species is by grouping them using shared traits. Trait-based approaches aid our mechanistic understanding of species biological responses to environmental change (Verberk et al. 2013). Having a diapause is one of these traits (Verberk et al. 2008; Tauber and Tauber 1981). Diapause, or temporary suspension of development, can be used by aquatic insects to escape unfavorable environmental conditions such as warming and drying caused by climate change. Diapause can occur in different life-stages and can be used to avoid stressful conditions (e.g. aestivation for avoiding adverse conditions in summer and hibernation for avoiding winter conditions). Many trichopteran species have adult diapause, where adults become comparatively inactive during summer and only oviposit towards autumn (this publication).

**Figure 1.**
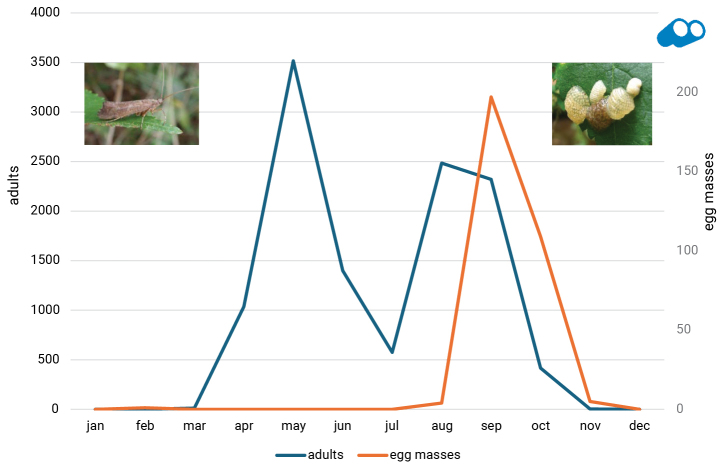
Phenology of adults and egg masses of *Glyphotaelius pellucidus* (Retzius, 1783) in the Netherlands (source: Observation.org). Data from 2005–2024. Plotted are the number of observations (which may pertain to more than 1 adult or egg mass).

In Trichoptera, species may diapause during the egg, larva, or adult stage. Diapause in the pupal stage is not currently known. Many Limnephilidae (and a single species of Phryganeidae) use a summer diapause in the adult stage as a means to aestivate. For example, consider the life cycle of *Glyphotaelius pellucidus* (Retzius, 1783), which was studied extensively by Kampwerth (2010). Females deposit eggs encapsulated in gelatinous matter on leaves in autumn. These leaves generally overhang a water body so when the larvae hatch (no egg diapause), the egg mass ruptures and the larvae drop into the water below and grow during the winter with pupation in early spring. Adults emerge in late spring and the flight period is not continuous consisting of two peaks: one in May and another in September with a reduction in flight over the summer (Fig. 1). This flight period could be erroneously interpreted as bivoltine. However, adults aestivate as a form of summer diapause, which explains the absence of summer flight activity. Eggs of this species, which are easily spotted, are seen only once in autumn (Fig. 1), providing evidence that the species is univoltine. If the species were bivoltine, eggs would appear twice each year.

During diapause, adults are less active and rarely recorded, as seen in *G. pellucidus* (Fig. 1). Some Limnephilidae species, such as *Stenophylax permistus* McLachlan, 1895 and *S. vibex* (Curtis, 1834), are known to shelter in caves (Leruth 1939, cited by Vergoossen and Hageman 2024), but for most species the exact refuges where diapausing adults remain hidden are unknown.

Adult diapause offers key advantages, particularly avoiding summer droughts. In autumn, adults can select oviposition sites that retain or will accumulate water, unlike species that aestivate as eggs or larvae and must choose sites before summer desiccation. This flexibility allows them to inhabit water bodies unsuitable for species with continuous aquatic larval stages.

With climate warming, seasonal water bodies are increasingly at risk of drying out. But also, when water bodies do not desiccate, the rise in water temperatures during summer presents problems for aquatic organisms, including aquatic trichopteran larvae, especially when combined with low oxygen conditions (Verberk et al. 2016). Species with diapausing adults may avoid harsh summer conditions in the water bodies, as (a large part of) the population spends the summer outside the water. Conversely, species without an adult diapause phase will have aquatic larvae during summer and are therefore expected to be more vulnerable to harsh summer conditions. From 2005 to 2024, the average water temperature in the Netherlands rose by 0.04 °C per year (https://www.clo.nl/indicatoren/nl022615-temperatuur-in-nederland-en-mondiaal-1907-2022). Also, the summers of 2018, 2019, 2020, and 2022 rank among the 5% driest summers that have ever occurred in the Netherlands. Given these changes, we expect that the proportion of species having an adult summer diapause will increase, while the proportion of non-diapausing species will decrease.

## ﻿Methods

### ﻿Dataset

**Figure 2.**
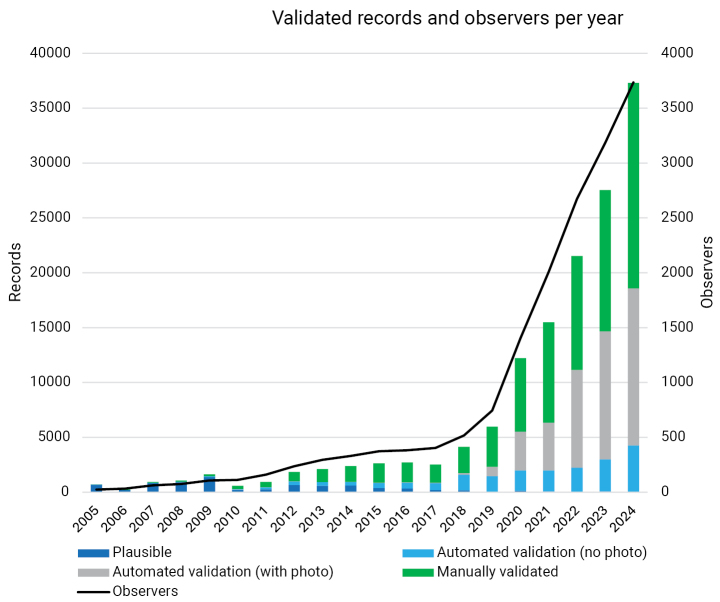
Number of validated records per year by validation category and numbers of observers per year.

**Figure 3.**
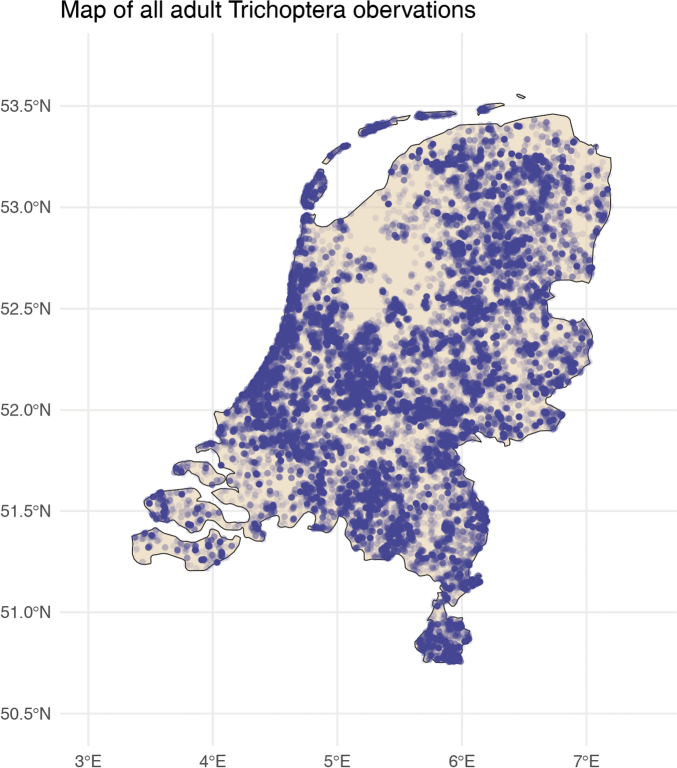
Map showing the geographic coordinates associated with the Trichoptera records in the Netherlands (in blue).

During 2005–2024, over 140 thousand records of Trichoptera or caddisflies, comprising 146 species, were submitted to the citizen-science portal Observation.org in the Netherlands. In the early years (2005–2010), numbers of records were modest. However, from 2019 onward, a strong increase is observed, with the total number of observers rising to 3,735 in 2024. The number of records per year and the number of observers are shown in Fig. 2. Most observers submitting records to the platform make casual observations rather than systematically collecting data. However, they explore a wide range of locations (Fig. 3). Many records come from gardens, both in urban and rural areas. Additionally, nature reserves attract many observers, leading to frequent recordings in these areas. Given this broad coverage, and adults being somewhat mobile, it is reasonable to assume that these data represent most aquatic habitats in the Netherlands.

### ﻿Selection of data and validation

Trichoptera sightings from 2005–2024 were downloaded from Observation.org (Tempelman et al. 2025). In total, 144,590 records were used in this analysis. The following selection criteria were applied:

1. **Year**: only data from 2005–2024 were included, as Observation.org was founded in 2005. Prior to that, only ~5,000 Trichoptera records were available.
2. **Stage**: only records of adult Trichoptera were selected.
3. **Taxonomic resolution**: Only records identified to species level were included; observations identified at genus level or higher were discarded.
4. **Validation**: only validated records were included. They break down into the following categories:

- **Accepted with evidence**: a manually validated photo by an Observation.org validator (**73,807 records**).
- **Accepted (plausible) – no photo**: submitted by a known and reliable observer (**7,885 records**). These include 1,462 records from Naturalis Biodiversity Centre (EIS-database) from **2005–2010.** These records were primarily collected as bycatch by lepidopterists and later identified by Dr. L.W.G. Higler, a renowned caddisfly expert. These records are considered reliable and assigned the validation status “accepted (plausible)”.
- **Automated validation (with photo)**: photos meeting a similarity threshold were validated by the automatic image recognition software (https://waarneming.nl/pages/disclaimer-obsidentify/), which is used by Observation.org (**43,958 records**).
- **Automated validation (no photo)**: a record without a photo was validated if a manually validated photo of the same species was taken in the same area and time frame (e.g. for *Glyphotaelius pellucidus*, within a 5 km radius and 1,000 days) (**18,939 records**).

In this study only the observations themselves were analyzed. The number of individuals observed was not taken into account. The latter number exhibits greater variability, especially for species with clumped distributions, such as Hydroptilidae. Furthermore, some observers do not register the number of individuals at all. Finally, a species’ abundance and the number of occurrences are often related, as seen in aquatic invertebrates (Verberk et al. 2010). So, it is also unlikely that trends reported here for number of observations would be very different if we had good data on species abundance.

Validated records submitted to Observation.org are regularly uploaded to the Global Biodiversity Information Facility (GBIF; https://www.gbif.org/), where they can be accessed via a GBIF account. The curated version of the data used for the analyses in this study have been made available separately in Tempelman et al. (2025).

### ﻿Species traits

A table was created listing all species that were recorded over 2005–2024. The following trait information was added to each species: diapause YES/NO, rarity of the species in the Netherlands and median length of the anterior wing. This information was based on Tempelman et al. (2022). The information on these traits, together with the number of records of adults of each trichopteran species, and the code used to analyse these data has been made available (Tempelman et al. 2025).

### ﻿Data analysis

We used binomial generalised linear mixed models (GLMs) to analyze changes in the number of occurrences (sightings) of a species, fitting the data with a binomial error distribution and a logit link function. These models treat each occurrence record of a given species in a given year (*n* = 144,590) as a separate observation.

Changes in species occurrence were analyzed using two approaches. First (Approach 1: summing species), we related the number of occurrences of a focal species each year to the total number of occurrences for that species across all years. Second (Approach 2: summing years), we related the number of occurrences of a given species in a specific year to the total occurrences for all species in that same year.

For example, in 2022, there were 1,635 records for *Glyphotaelius pellucidus* out of a total of 21,535 records for that year. Over all years, there were 12,137 records for *G. pellucidus*. In Approach 1, the 1,635 records for *G. pellucidus* were compared to the remaining 10,502 records from other years. In Approach 2, the same 1,635 records from 2022 were compared to the remaining 19,900 records in 2022 belonging to other species.

In both approaches, the binomial GLMs included diapause presence/absence, species wing size, and national rarity as fixed factors. To visualize the model outputs, we plotted model predictions. In the same figure (for illustrative purposes only), we also included the fractions of occurrence records, which summed to 1 either across all years for a given species (Approach 1) or across all species for a given year (approach 2).

All analyses were conducted in R Statistical Software v. 4.4.2 (R Core Team 2024) and our code is publicly available (Tempelman et al. 2025).

## ﻿Results

### ﻿Summing species

**Figure 4.**
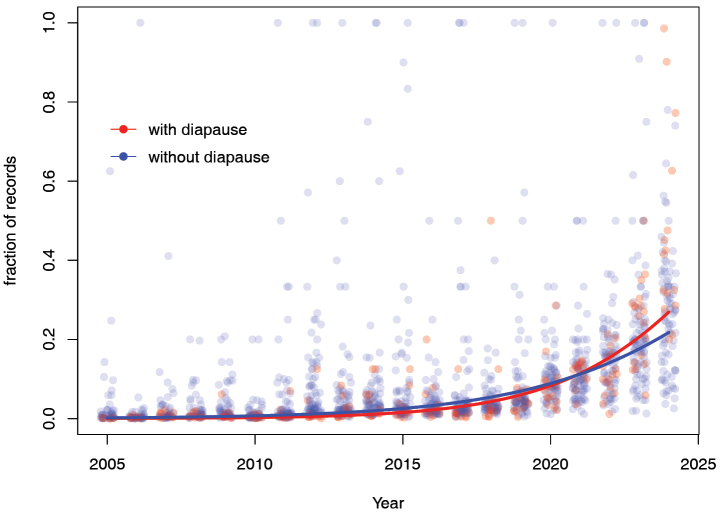
Increase in number of records, split into species with diapause (shown in red) and without diapause (shown in blue). The points indicate the fraction per species, per year, which over the years adds up to 1 for each species. The lines show the model fits over an average for all species of a given diapause category.

As a result of the increasing use of Observation.org by more and more observers, a growing number of sightings was recorded over the time period for nearly all species. This is also reflected in the first approach where fractions per species accumulated to 1 across all years (Fig. 4). Although species are recorded more frequently in recent years, a significant interaction between adult diapause and year was found in this analysis (z = −43.60; *P* <0.001; Table 1), with the number of sightings increasing more rapidly for species that have an adult summer diapause when compared to those without diapause (termed non-diapausing species hereafter) (Fig. 4).

**Table 1.** Model summary for species fractions. Species commonness is ranked from 0 (most rare) to 4 (most common). Note that the commonness of species was not included as a main factor in the model as the analysis focused on the species fractions, which already accounts for differences in the commonness and rarity of species.

Term	Estimate	Std. Error	z value	Pr(>|z|)
Intercept (species with diapause)	−737.00	6.96	−105.9	<0.0001
Year (Y)	0.364	0.00344	105.6	<0.0001
Wing length (WL)	2.83	0.479	5.91	<0.0001
Species without diapause	177.11	4.062	43.60	<0.0001
Y x WL	−0.00140	0.000237	−5.91	<0.0001
Y x commonness	−0.00000791	0.0000015	−5.27	<0.0001
Y:Species without diapause	−0.0876	0.00201	−43.60	<0.0001

**Figure 5.**
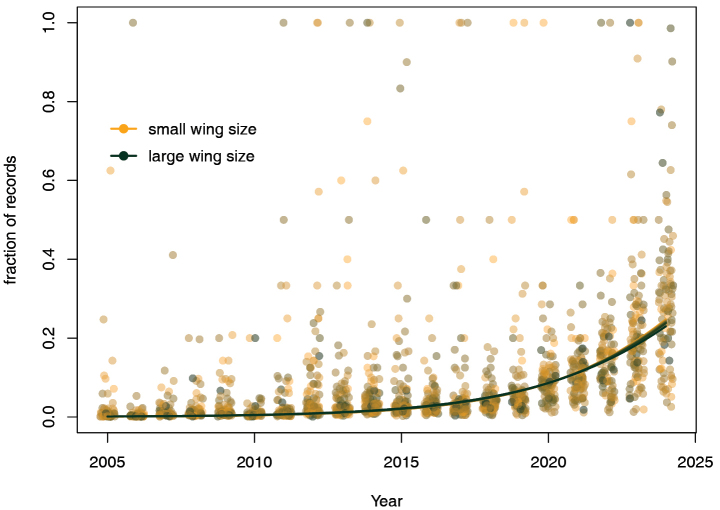
Increase in number of observations, broken down by mean length of the anterior wing. The points indicate the fraction per species, per year, which over the years adds up to 1 for each species. Lines show the model fits for different percentiles (0^th^, 10^th^, 20^th^, up to 100^th^ percentile), with smaller wings shown in yellow and larger wings shown in green.

Also, slight differences were found in trends between species as a function of their wing size (Fig. 5; z = –5.91; *P* <0.001; Table 1) and rarity (z = −5.27; *P* < 0.001; Table 1); but in both cases, their effect was much smaller than the difference with adult diapause.

### ﻿Summing years

**Figure 6.**
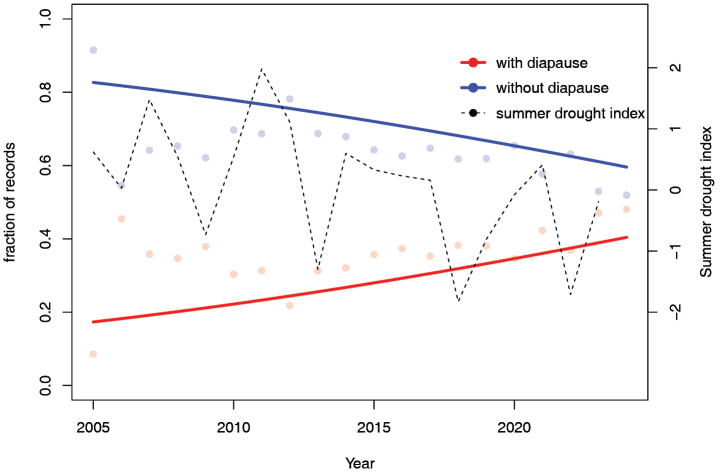
Sum of fractions of number of observations, added up for diapausing species (shown in red) and non-diapausing species (shown in blue). The points indicate the fraction per year, which adds up to 1 across all species. Points show the cumulative fraction for a given year across all species, while lines show the model fits. The dotted line represents the Standardized Precipitation–Evaporation Index (SPEI) or Summer drought index (https://www.knmi.nl/kennis-en-datacentrum/achtergrond/achtergrondinformatie-neerslagindex-spi). In this index, negative values indicate drier conditions.

When analysing the data by yearly fractions, there is a clear increase in the proportion of species with a diapause, while species without a diapause decrease in proportion (z = –38.84; *P* <0.001; Table 2; Fig. 6), resembling the pattern found for the species fractions (Fig. 2). Furthermore, common species were recorded more often than rare species in recent years (z = 19.15; *P* < 0.001; Table 2).

**Table 2.** Model summary for yearly fractions. Note that the interaction between year and the commonness of species was not significant and therefore dropped from the model.

Term	Estimate	Std. Error	z value	Pr(>|z|)
Intercept (species with diapause)	−7.95	8.07	−0.985	0.324
Year (Y)	0.000282	0.00399	0.0708	0.944
Wing length (WL)	1.707	0.413	4.134	<0.0001
Species without diapause	133.0	3.47	38.31	<0.0001
Commonness of species (CR)	−27.41	1.48	−18.48	<0.0001
Y x WL	−0.000824	0.000204	−4.037	<0.0001
Y:Species without diapause	−0.0665	0.00171	−38.84	<0.0001
Y x CR	0.0141	0.000734	19.14	<0.0001

## ﻿Discussion

The increase in the usage of Observation.org was demonstrated by the general increase in the number of sightings, which rose from several hundreds of records to over 35,000 records in 2024 (Fig. 2). Interestingly, this increase in sightings differed across species. Not surprisingly, larger species and those with distinctive wing markings are easier to notice and hence their number of sightings increased disproportionally. However, the effect of body size was small compared to the effect of diapause. Sightings for species with a summer diapause also increased disproportionally. Since the rarity and body size of species was considered, the effect of diapause vs non-diapausing individuals is genuine rather than confounded by the ease with which a given species can be observed.

As Trichoptera are dependent on water during their larval periods, the amount of rain and length of dry periods are likely significant in explaining the patterns documented here. In recent years, several hot and dry summers occurred in the Netherlands (see Introduction), which is likely to negatively impact species that have summer larvae, such as *Mystacides longicornis* (Linnaeus, 1758) and others without diapausing adults. Even if aquatic habitats do not dry out, they may reach high temperatures, rising over 30 °C, which is near the lethal limits recorded for aquatic invertebrates with aquatic respiration, especially when considering exposure durations for several hours or days (Verberk et al. 2023). Conversely, species that lack larval life stages in summer and also exhibit adult summer diapause, such as *Glyphotaelius pellucidus*, can retreat to cooler habitats and circumvent the harsh conditions during summer, which explains the increased number of sightings of diapausing species. In the future, it would be useful to determine whether the presence of species with or without adult summer diapause is related to annual differences in weather conditions, such as the occurrence of dry summers in the previous year (one approach could be to relate these occurrences to a drought index, as in Fig. 6) to help validate some of the points discussed above.

Citizen scientists increasingly generate data on species occurrences, and these data may be useful to climate scientists interested in tracking species-level population and demographic trends. These volunteers have been shown to sample different types of habitats with different collection methods compared to professional scientists, thus providing potential complementary, useful information to workers (Peeters et al. 2022). Identification of freshwater invertebrates correctly is challenging for amateur naturalists and collectors, and caddisflies are no exception. A recent study comparing data collected on aquatic invertebrates by either volunteers or professionals showed that volunteers tend to make less complete inventories compared to professionals, but they were able to sample a much greater number of locations (Peeters et al. 2022). Submitting observations to the citizen-science portal Observation.org requires pictures to be uploaded and these photographs are verified by experts. By doing so, citizen scientists are able to collect high quantities of presence-only data on Trichoptera. There is strength in numbers; after such data are rigorously verified and validated, they become a valuable resource for scientists.

## ﻿Conclusions

Over 2005–2024, the proportion of Trichoptera species that have a summer diapause increased, while the proportion of species without a summer diapause decreased.

Dry summers in previous years may be a driver for these changes in proportion to species with and without diapause.

Adult Trichoptera occurrence data collected by citizen scientists may help in understanding changes to population and distribution patterns, which may be related to climate change.

## ﻿Recommendations

Future research should focus on improving our understanding of how species respond to changing climatic conditions by directly relating observations of Trichoptera adults to weather conditions such as temperature and precipitation.

Promoting citizen-science initiatives to collect extensive Trichoptera population data, along with educational campaigns, will enhance both public engagement and data accuracy.

## ﻿Additional information

### Use of AI

Utilized artificial intelligence (AI) tools was used to assist in the preparation of this manuscript. Specifically, ChatGTP was employed to help draft and refine sections such as the abstract, ensuring it accurately summarized the study’s findings and communicated them effectively. Additionally, AI provided feedback on code used to analyse the data. We found ChatGPT to be a valuable collaborator for brainstorming ideas and hope it gained an appreciation for caddisflies in the process.

### Author ORCIDs

Wilco C. E. P. Verberk https://orcid.org/0000-0002-0691-583X

### Data availability

All the data and the code used to analyse them, supporting the findings of this study, are available in: Tempelman et al. (2025).
